# Immunolocalization of Arthropsin in the Onychophoran *Euperipatoides rowelli* (Peripatopsidae)

**DOI:** 10.3389/fnana.2016.00080

**Published:** 2016-08-04

**Authors:** Isabell Schumann, Lars Hering, Georg Mayer

**Affiliations:** ^1^Department of Zoology, Institute of Biology, University of Kassel, KasselGermany; ^2^Molecular Evolution and Animal Systematics, University of Leipzig, LeipzigGermany

**Keywords:** onychophora, opsins, central nervous system, mushroom bodies, olfactory system

## Abstract

Opsins are light-sensitive proteins that play a key role in animal vision and are related to the ancient photoreceptive molecule rhodopsin found in unicellular organisms. In general, opsins involved in vision comprise two major groups: the rhabdomeric (r-opsins) and the ciliary opsins (c-opsins). The functionality of opsins, which is dependent on their protein structure, may have changed during evolution. In arthropods, typically r-opsins are responsible for vision, whereas in vertebrates c-opsins are components of visual photoreceptors. Recently, an enigmatic r-opsin-like protein called arthropsin has been identified in various bilaterian taxa, including arthropods, lophotrochozoans, and chordates, by performing transcriptomic and genomic analyses. Since the role of arthropsin and its distribution within the body are unknown, we immunolocalized this protein in a representative of Onychophora – *Euperipatoides rowelli* – an ecdysozoan taxon which is regarded as one of the closest relatives of Arthropoda. Our data show that arthropsin is expressed in the central nervous system of *E. rowelli*, including the brain and the ventral nerve cords, but not in the eyes. These findings are consistent with previous results based on reverse transcription PCR in a closely related onychophoran species and suggest that arthropsin is a non-visual protein. Based on its distribution in the central brain region and the mushroom bodies, we speculate that the onychophoran arthropsin might be either a photosensitive molecule playing a role in the circadian clock, or a non-photosensitive protein involved in olfactory pathways, or both.

## Introduction

In bilaterians, opsin proteins are essential molecules for mediating the ability to detect and use light for diverse biological functions, including color vision (reviewed by Porter et al., 2011). These proteins are members of the superfamily of G-protein coupled receptors with a seven transmembrane α-helical structure (e.g., Foster and Bellingham, 2002; Henze et al., 2012; Hering and Mayer, 2014; Yan et al., 2014). G-protein coupled receptors comprise the largest group of proteins in animal genomes and its members are able to detect signaling molecules such as hormones, neurotransmitters, and odorants (Park et al., 2013). Therefore, these receptors are involved in a variety of processes and signaling cascades, including chemo- and photoreception (e.g., Hill et al., 2002; Ronnett and Moon, 2002; Liang et al., 2003; Ache and Young, 2005; Nilsson, 2009). Together with a light-sensitive, vitamin A-derived prosthetic group as chromophore – the retinal – opsin proteins build the photoreceptive molecule rhodopsin, which is involved in various visual and non-visual signaling functions, such as chemoreception or circadian clocks (e.g., Terakita, 2005; Gregory, 2008; Porter et al., 2011; Oakley and Speiser, 2015). Furthermore, opsin proteins are characterized by the amino acid lysine in the seventh position of the transmembrane helix (Vopalensky and Kozmik, 2009). The lysine serves as an attachment site for the chromophore (Cronin et al., 2010). In this assemblage, the chromophore triggers specific phototransduction cascades associated with animal vision or circadian clocks by changing its conformation from 11-*cis* to all-*trans*-retinal (e.g., Fernald, 2000; Kashiyama et al., 2009; Gehring and Seimiya, 2010; Hering and Mayer, 2014; Liegertová et al., 2015).

Besides their occurrence in photoreceptors used for vision, opsins have been discovered in a wide variety of tissues and cell types (Liegertová et al., 2015). Studies on the sea urchin *Strongylocentrotus purpuratus* revealed expression of distinct opsins in the pedicellariae and tube feet, although these structures have not been demonstrated to act as light-sensitive organs thus far (Raible et al., 2006). Also in the jellyfish *Tripedalia* sp. six out of 18 opsins occur in the eye, whereas the remaining opsins are expressed in the tentacles, manubrium, and other tissues (Gehring and Seimiya, 2010). Consequently, opsin-dependent functions are not restricted to the eyes but occur also in other tissues, including the central nervous system (e.g., Terakita et al., 2012; Eriksson et al., 2013). Some opsin-dependent pathways might indeed play a role in molecular mechanisms other than photosensitivity; for example, it has been shown recently that opsin proteins are able to serve as olfactory receptors (Terakita et al., 2012; Park et al., 2013).

Opsins are ancient molecules that have diverged before the deuterostome-protostome split (Oakley and Speiser, 2015). Subsequently, opsins have gone through multiple duplications during evolution (Koyanagi et al., 2008; Suga et al., 2008). The last common ancestor of bilaterians most likely had six opsins, including two rhabdomeric opsins, one ciliary opsin, three Group 4 opsins and one Go opsin. Within the bilaterians, three major groups of opsins have been classified: ciliary, rhabdomeric, and retinal G protein-coupled receptor opsins (Foster and Bellingham, 2002; Arendt et al., 2004; Nilsson, 2005; Plachetzki et al., 2007; Feuda et al., 2012; Hering and Mayer, 2014). The rhabdomeric opsins (r-opsins) are responsible for photoreception in arthropods, whereas the ciliary opsins (c-opsins) are used for vision in vertebrates (Suga et al., 2008; Backfisch et al., 2013).

**FIGURE 1:**
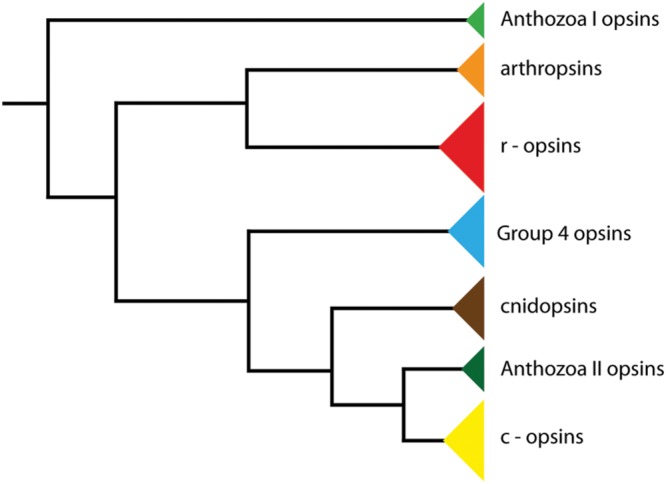
**Phylogeny of opsin genes.** The tree illustrates the position of arthropsins as the sister group of r-opsins, including melanopsins (phylogenetic relationships according to Hering and Mayer, 2014).

The last common ancestor of Panarthropoda (Onych ophora + Tardigrada + Arthropoda), on the other hand, possessed one c-opsin, two r-opsins and two Group 4 opsins (Hering and Mayer, 2014). So far, three opsins have been identified in onychophorans: a non-visual c-opsin, a visual r-opsin (onychopsin), and a second putative r-opsin, the so-called arthropsin (Hering et al., 2012; Eriksson et al., 2013). Arthropsins are a set of potential r-opsins that were first discovered in the genome of the crustacean *Daphnia pulex* (see Colbourne et al., 2011). Putative arthropsin homologs have also been identified in the annelids *Capitella teleta* and *Platynereis dumerilii*, the molluscs *Lottia gigantea* and *Crassostrea gigas*, the wandering spider *Cupiennius salei*, the pea aphid *Acyrthosiphon pisum*, several dragonfly species, and the onychophoran *Euperipatoides kanangrensis* (see Hering et al., 2012; Eriksson et al., 2013; Hering and Mayer, 2014; Futahashi et al., 2015). Phylogenetic analyses revealed the arthropsin clade as the sister group of the bilaterian r-opsins, including melanopsins (**Figure 1**), (Hering et al., 2012; Hering and Mayer, 2014). Furthermore, reverse transcription PCR data have indicated that arthropsin might be expressed only in the central nervous system, whereas onychopsin (r-opsin) and c-opsin occur in the eyes and brain tissue of the onychophoran *E. kanangrensis* (see Eriksson et al., 2013). It remains unknown, however, in which part of the onychophoran nervous system arthropsin is localized.

So far, only a few studies have been carried out on arthropsins. An analysis of the expression level of arthropsin in the arthropod *Sympetrum frequens* and other dragonflies revealed that this protein is undetectable in the adult eye (Futahashi et al., 2015). Although this enigmatic protein occurs in various bilaterians, neither immunolocalization nor functional data are available. The major objective of our study is therefore to localize arthropsin in a representative of Onychophora (velvet worms), *Euperipatoides rowelli*, by antibody labeling.

## Materials and Methods

### Specimen Collection and Maintenance

Specimens of *Euperipatoides rowelli* Reid, 1996 (Onychophora, Peripatopsidae) were collected from decaying logs in the Tallaganda State Forest (New South Wales, Australia, 35°26′S, 149°33′E, 954 m) in October 2011, and January 2013. Permission for specimen collection was obtained from the Forestry Commission of New South Wales (permit no. SPPR0008). The animals were kept in plastic jars with perforated lids at 18°C as described by Baer and Mayer (2012) and fed with frozen crickets (*Acheta domesticus*) every 4 weeks. One-year-old specimens of *E. rowelli* were used for all experiments.

### Antibody Generation

Polyclonal antibodies against HPLC-purified synthetic peptides of arthropsin from *E. rowelli* were newly generated following coupling to KLH (key limpet hemocyanin; Peptide Specialty Laboratories GmbH, Heidelberg, Germany). Anti-arthropsin antibodies (antigen: C-KYFGHSKQWSSSYSTVRDWTSSR) were purified from sera of immunized guinea pigs (Peptide Specialty Laboratories GmbH).

### Immunolocalization on Vibratome Sections

For immunolabeling on Vibratome sections, heads of specimens of *E. rowelli* (*n* = 25) were fixed overnight in 4% paraformaldehyde (PFA) in phosphate-buffered saline (PBS, 0.1 M, pH 7.4) at 4°C, embedded in a 4:1 mixture of albumin/gelatin and fixed again overnight in 10% PFA in 0.1M PBS (pH 7.4) at 4°C (Oliveira et al., 2013). The fixed albumin/gelatin blocks were washed for 30 min in 1% PBS-Tx (Triton^®^ X-100; pH 7.4) and sectioned into series of 100–150 μm thin sections with steel blades on a Vibratome (Vibratome Company, Saint Louis, MO, USA). The sections were washed several times with 1% PBS-Tx and blocked in 1% bovine serum albumin (BSA) in 0.1 M PBS (pH 7.4) for 60–90 min. Incubations with the primary antibody (guinea pig anti-arthropsin [0.7 mg/ml], diluted 1:50 or 1:500 in 0.1 M PBS) were carried out overnight at room temperature by gentle shaking, followed by 2 days at 4°C without shaking. After additional washing steps with 0.1 M PBS, the sections were incubated with the secondary antibody (Alexa Fluor^®^ 488 donkey anti-guinea pig IgG, Dianova, Hamburg, Germany, diluted 1:500 in 0.1 M PBS) for another 2 days at room temperature by gentle shaking. The sections were washed several times with 0.1 M PBS (pH 7.4), stained with propidium iodide (Invitrogen, Carlsbad, CA, USA; DNA marker, diluted 1:6000 in 0.1 M PBS) or TO-PRO^®^-3 idodide (Invitrogen, diluted 1:6000 in 0.1 M PBS) for 15 min, mounted on slides in Vectashield (H-1000, Vector Laboratories Inc., Burlingame, CA, USA) and stored at 4°C in darkness.

### Testing Antibody Affinity to Er-arthropsin

For western blots on tissue lysate, the brains of *E. rowelli* (*n* = 2) were dissected in 0.1 M PBS (pH 7.4) and incubated in a protein extraction buffer, after which they were treated with liquid nitrogen and then ultra-sonicated to bring the proteins into solution. Before blotting, different dilutions of protein extract were loaded in 1x Laemmli buffer and dithiothreitol (DTT) on a 12% sodium dodecyl sulfate (SDS) polyacrylamide gel and run for 2 h at 100 V. Then the SDS gel were blotted for 60 min at 100 V on a nitrocellulose membrane in Towbin transfer buffer, washed a few minutes in Tris-buffered saline with 1% Tween-20^®^ wash buffer (TBS-T) and blocked with blocking buffer (5% skimmed milk powder [=SMP] in 1x TBS-T). The primary antibody (guinea pig anti-arthropsin, diluted 1:500 in 1x TBS-T-SMP) was added to the shrink-wrapped membrane and incubated overnight at 4°C by gentle shaking. Following several washing steps (5 min) with 1x TBS-T, the secondary antibody (anti-guinea pig horseradish peroxidase, dilution 1:5000) was added to the membrane and incubated for 2 h at room temperature by gentle shaking. For chemiluminescent reaction, the membrane was incubated for 5 min in a home-made chemiluminescent reagent (ECL; contains 66 μl cumarin, 150 μl luminol, and 10.1 μl 30% hydrogen peroxide in 30 ml Tris-HCl pH 9.5) and visualized with myECL^TM^ Imager (Life Technologies GmbH, Darmstadt, Germany).

In addition, western blots were carried out on the recombinant expressed protein Er-arthropsin. Since the recombinant protein was predicted to be insoluble when overexpressed in *Escherichia coli*^1^ (see Diaz et al., 2009) we decided to use a truncated version of the protein in this experiment, which comprises the C-terminal-most region (181 of 419 amino acids) of the protein, including the anti-arthropsin antigen. Thus, the target fragment was cloned into the pMAL-c2 vector system (NEB, Ipswich, MA, USA) and fused to maltose-binding protein (MBP), which has been shown to enhance the solubility of the target protein (Kapust and Waugh, 1999). Thereby, total RNA from a male specimen of *E. rowelli* was extracted and purified using TRIzol Reagent (Life Technologies, Carlsbad, CA, USA) and RNeasy MinElute Cleanup Kit (Qiagen, Hilden, Germany) and first strand cDNA was reverse transcribed using random hexamer primers and SuperScript IV Reverse Transcriptase (Life Technologies) according to the manufacturers’ protocols. Using Phusion High-Fidelity DNA Polymerase (Thermo Scientific, Waltham, MA, USA) and the first strand cDNA as template, a 546 bp-long fragment of *Er-arthropsin* was amplified using gene specific forward (5′-gcggatccATGGCAGAAACTCTTGCTAATTC-3′) and reverse primers (5′-gcgtcgacTTATATTATATTTATTTTCTTATCCACCTTGG-3′), which contained restriction sites (forward: BamHI, reverse: SalI) that were required for subsequent cloning into the pMAL-c2 vector to generate the plasmid pMAL-Er-arthropsin. The pMAL-Er-arthropsin plasmids were transformed into *Escherichia coli* PC2889 cells. Cells were grown in 100 ml LB medium at 37°C until the OD_600_ of 0.4 was reached. IPTG (0.5 mM final concentration) was added and the cells were further cultured for 2.5 h at 37°C before harvesting. From this bacterial suspension, 1 ml was taken, centrifuged at 4000 × *g* for 15 min and the pellet lysed in 2x Laemmli buffer containing protease inhibitor tablets (Roche, Basel, Switzerland), heated to 95°C for 10 min and stored at -20°C until further use. The remaining bacterial suspension was centrifuged at 4000 × g for 15 min. The pellet was washed and resuspended in buffer A (20 mM Tris-HCl pH 7.4, 200 mM NaCl, 1 mM EDTA). After sonication (UP 200s; Hielscher Ultrasonics GmbH, Teltow, Germany), the solution was centrifuged at 10000 × g for 20 min. Six volumes of buffer A were added to the supernatant and loaded onto an amylose resin column (NEB) for purification of the MBP-fused arthropsin. The column was washed with 12 volumes of buffer A and afterward the purified protein was eluted in 500 μl buffer A containing 20 mM maltose. Both, the lysate from the bacterial suspension as well as the amylose-purified protein (at a concentration of 0.2 mg/ml) were loaded and run for 1 h at 200 V on six 12% SDS polyacrylamide gels. Then the SDS gels were blotted for 45 min at 150 V on nitrocellulose membranes (Porablot NCP; Macherey-Nagel, Düren, Germany) and blocked with blocking buffer (4% skimmed milk powder in 1x PBS) for 30 min. For blocking experiments with an excess of the antigen peptide used for the generation of the antibody, the primary antibody (guinea pig anti-arthropsin, 0.7 mg/ml) was incubated with twice (2x) as well as 10-fold (10x) the amount of the synthetic peptide for 1 h at room temperature and added to the six membranes at the following concentrations: unblocked antibody, 1:50; blocked antibody (2x), 1:50; blocked antibody (10x), 1:50; unblocked antibody, 1:500; blocked antibody (2x), 1:500; blocked antibody (10x), 1:500. The six membranes were incubated overnight at room temperature with gentle shaking. Following six washing steps (5 min each) with 1x PBS, the secondary antibody (anti-guinea pig alkaline phosphatase, dilution 1:10000; Dianova) was added to the membrane and incubated for 3 h at room temperature with gentle shaking. For staining reaction, the membranes were washed with AP buffer (contains 100 mM NaCl, 5 mM MgCl_2_, 100 mM Tris-HCl pH 9.5) and incubated for 1 h with AP buffer + BCIP.

### Confocal Microscopy, Light Microscopy, and Image Processing

Vibratome sections were analyzed with the confocal laser-scanning microscope Leica TCS STED (Leica Microsystems, Wetzlar, Germany). The images were processed with Adobe (San Jose, CA, USA) Photoshop CS5.1 and Leica LAS AF Lite (Leica Microsystems). Final panels and diagrams were designed using Adobe (San Jose, CA, USA) Photoshop CS5 and Illustrator CS5.

## Results

### Immunolocalization of Arthropsin in *Euperipatoides rowelli*

**FIGURE 2:**
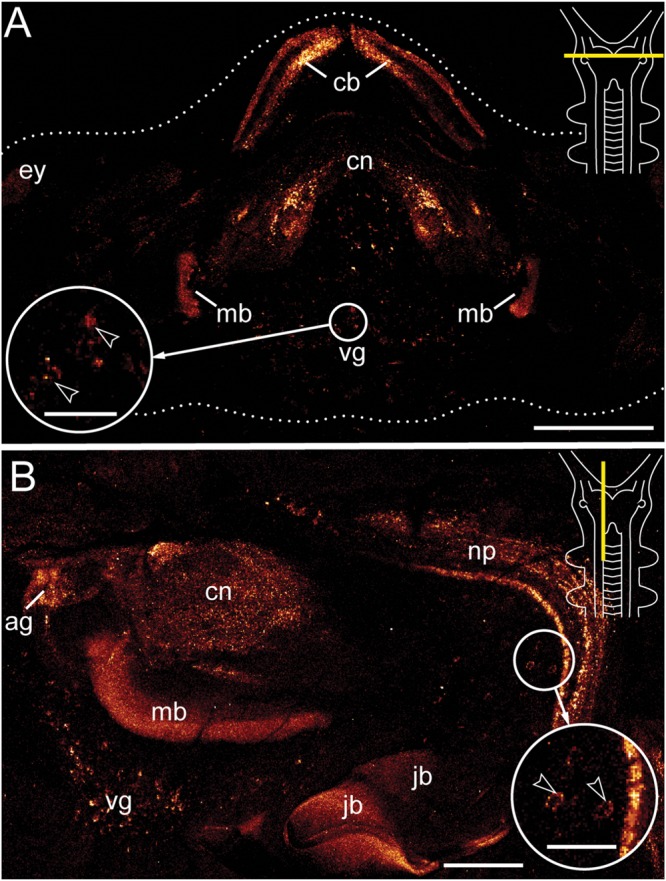
**Immunolocalization of arthropsin in the nervous system of the onychophoran *Euperipatoides rowelli*.** Vibratome sections of heads. Confocal micrographs. Dorsal is up in all images. Insets in the upper right corners illustrate sectional plates (yellow bars). **(A)** Cross section. Note the presence of immunoreactive fibers in the central body, the central neuropil, and the inner lobes of the mushroom body. Note also a ventral group of immunoreactive somata within the protocerebrum (inset, arrowheads). **(B)** Sagittal section. Immunoreactive fibers are present in the antennal glomeruli of the protocerebrum and the connecting cord that links the ventral nerve cord to the brain. Note also the neuronal somata associated with the connecting cord (inset, arrowheads). The jaw blades (jb) are autofluorescent. Abbreviations: ag, antennal glomeruli; cb, central body; cn, central neuropil; ey, position of the eye; jb, jaw blades; mb, mushroom body; np, neuropil of the connecting cord; vg, ventral group of cells. Scale bars: 250 μm **(A)**, 125 μm **(B)**, 50 μm (insets **A,B**).

**FIGURE 3:**
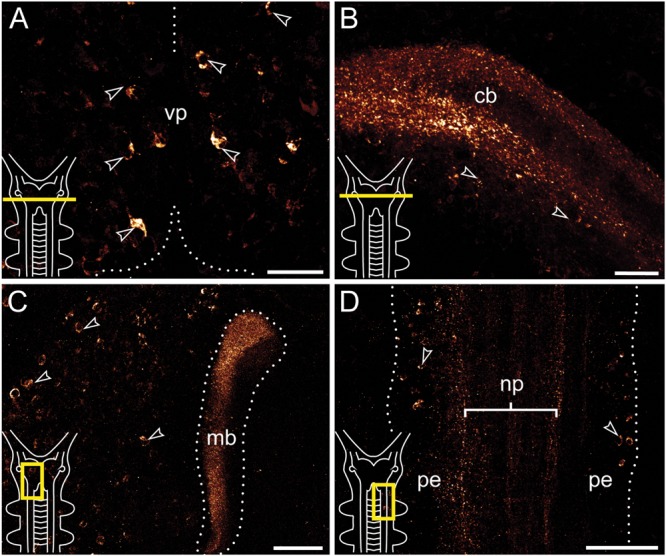
**Immunolocalization of arthropsin in the nervous system of the onychophoran *Euperipatoides rowelli* Vibratome sections.** Confocal micrographs. Insets in the lower left corners illustrate sectional planes (yellow bars). **(A)** Cross section of the ventral portion of the brain. Dorsal is up. Note the ventral group of somata in the protocerebrum (arrowheads). **(B)** Cross section of the central body. Dorsal is up. Note the outer and inner fiber layers and the associated immunoreactive somata (arrowheads). **(C)** Horizontal section of the mushroom body. Anterior is up, median is left. Note prominent immunoreactivity in the inner lobe of the mushroom body and numerous immunoreactive somata in the medioventral region of the protocerebrum (arrowheads). **(D)** Horizontal section of the ventral nerve cord. Anterior is up, median is left. Note immunoreactive fiber bundles within the nerve cord neuropil with associated somata in the perikaryal layer (arrowheads). Abbreviations: cb, central body; mb, mushroom body; np, nerve chord neuropil; pe, perikaryal layer; vp, ventral protocerebrum. Scale bars: 25 μm **(A,B)**, 50 μm **(C)**, 100 μm **(D)**.

**FIGURE 4:**
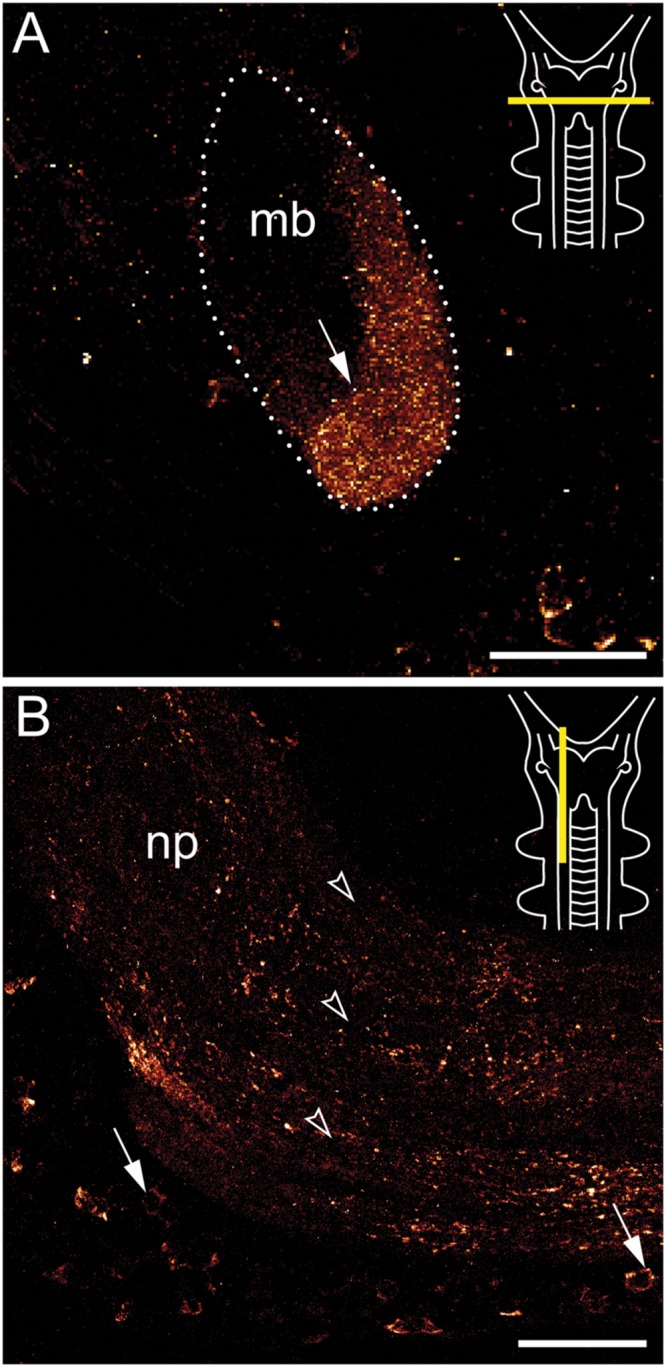
**Immunolocalization of arthropsin in the nervous system of the onychophoran *Euperipatoides rowelli.*** Vibratome sections of heads. Confocal micrographs. Dorsal is up in all images. Insets in the upper right corners illustrate the position of sectional planes (yellow bars). **(A)** Cross section of the mushroom body. Note the presence of immunoreactive fibers in the inner/median lobe (arrow). **(B)** Sagittal section of anterior nerve cord. Note the immunoreactive somata (arrows) within the perikaryal region and fibers within the nerve cord neuropil (arrowheads). Abbreviations: mb, mushroom body; np, nerve cord neuropil. Scale bars: 50 μm **(A,B)**.

**FIGURE 5:**
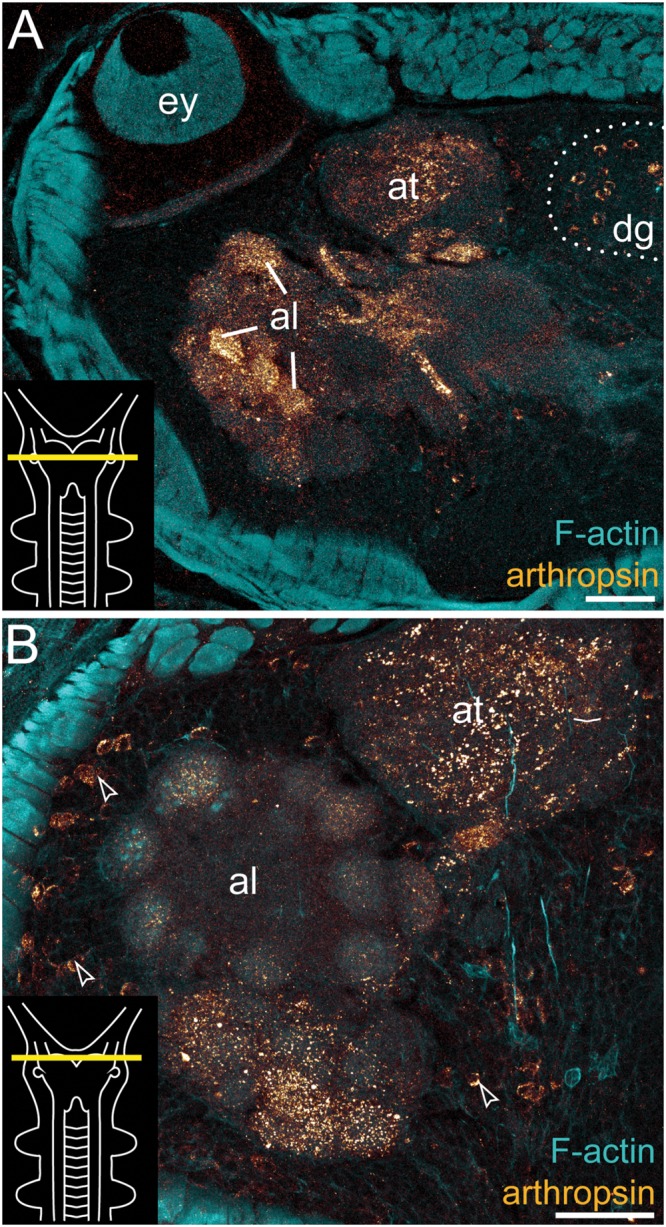
**Immunolocalization of arthropsin in the nervous system of *Euperipatoides rowelli*.** Vibratome cross sections of heads. Confocal micrographs. Double-labeling with anti-arthropsin antibody (glow-mode) and phalloidin-rhodamine (cyan). Dorsal is up in all images. Insets in the lower left corners illustrate sectional planes (yellow bars). **(A)** Detail of a cross section at the level of the eye. Note numerous immunoreactive fiber bundles in the antennal glomeruli and the antennal tract and the complete lack of immunoreactivity within the eye. Note also a dorsal group of immunoreactive somata (dotted line). **(B)** Detail of the brain region containing the antennal lobe. Note immunoreactive fibers in the antennal lobe and adjacent somata of immunoreactive neurons (arrowheads). Abbreviations: al, antennal lobe; at, antennal tract; dg, dorsal group of somata (encircled by a dotted line); ey, eye. Scale bars: 75 μm **(A)**, 50 μm **(B)**.

Antibody labeling against Er-arthropsin revealed immuno reactive fibers and somata of neurons in the central nervous system, specifically in the brain and the ventral nerve cords (**Figures 2–5**). Within the brain, two expressing groups of somata are found in each brain hemisphere: a dorsal group, which lies posterior to the central body, and a ventromedian group with a higher number of somata that are larger than those of the dorsal group (**Figures 2** and **3A,C**).

Immunoreactive fibers are present in all major brain neuropils, including the central body and the mushroom bodies. Within the central body, immunoreactivity occurs mainly in the dorsal and ventral layers (**Figures 2A** and **3B**). A dense network of fibers is also present in the central brain neuropil linking the two brain hemispheres (**Figure 2B**). Prominent immunoreactive fibers further occur within each mushroom body, in which they are restricted only to the median longitudinal lobes (**Figures 2A**, **3C**, and **4A**).

Additional fibers are found in the antennal lobes, including the olfactory glomeruli, as well as in the antennal tract (**Figures 2B** and **5**). The antennal lobes are associated with immunoreactive somata that are adjacent to the outer olfactory glomeruli (**Figure 5B**). The two ventral nerve cords and the connecting cords (=anterior-most medullary portions of the central nervous system linking the brain to the ventral cords) also contain a high number of immunoreactive fibers and somata (**Figures 2B**, **3D**, and **4B**). While the immunoreactive fibers are condensed to prominent bundles within the neuropil of each nerve cord, single somata are distributed in the perikaryal layer along each nerve cord and do not show any conspicuously ordered or condensed arrangement (**Figure 3D**).

Notably, despite the high number of immunolabeled specimens of *E. rowelli* (*n* = 25), we found no fibers or somata of immunoreactive neurons in the optic tract or photoreceptor layer of the eye (**Figure 5A**). Non-specific signal (due to autofluorescence; cf. Figure 9 in Oliveira et al., 2013 and Figure 4.19 in Mayer et al., 2015) occurs in the sclerotized cuticle of each jaw blade within the mouth (**Figure 2B**)

### Testing the Affinity of Anti-arthropsin Serum to Er-arthropsin

**FIGURE 6:**
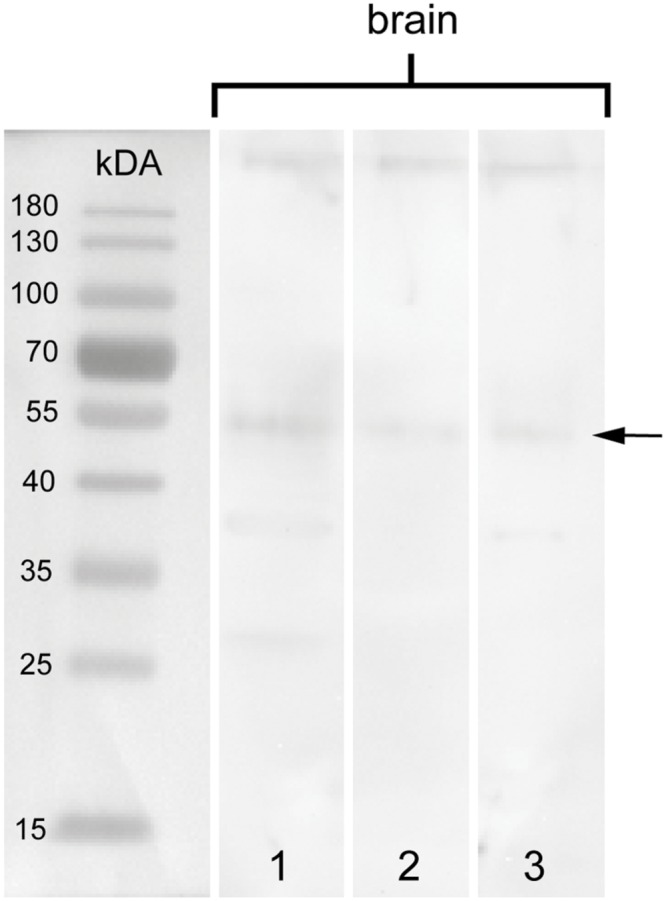
**Western blot of arthropsin antisera using brain tissue of *Euperipatoides rowelli*.** Different lanes contain different dilutions of protein extract. Lane 1: Supernatant of extracted proteins (35.84 μg). Lane 2: Supernatant of extracted proteins at lower concentration (17.92 μg). Lane 3: Resuspended pellet in protein extraction buffer (46.787 μg). Note a distinct band at 55 kDA in all lanes (arrow).

**FIGURE 7:**
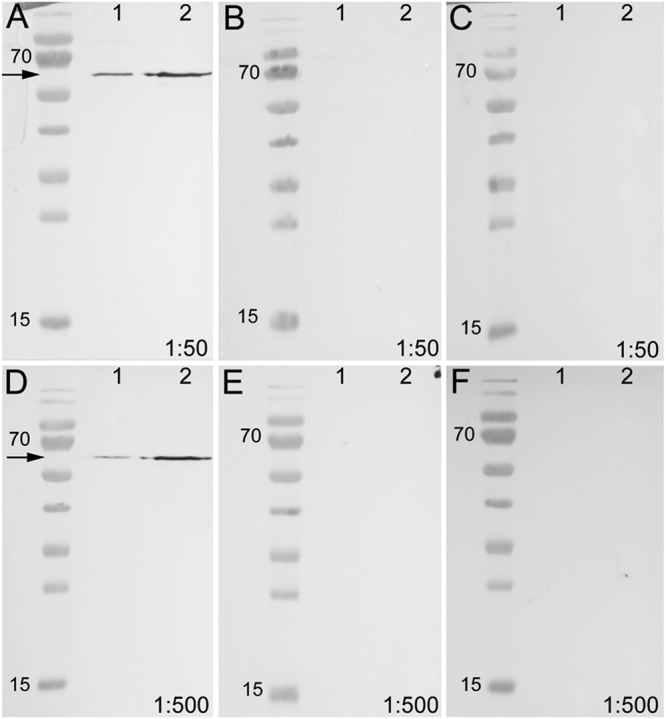
**Western blot of unblocked and blocked arthropsin antisera using recombinant expressed protein extracted from *Escherichia coli*.** Truncated Er-arthropsin (20 kDa) fused with MBP (42,5 kDa) was used for all experiments. Lane 1 was loaded with the bacterial cell lysate of *Escherichia coli* and lane 2 with the purified Er-arthropsin-MBP fusion protein in all images. **(A)** Unblocked anti-arthropsin antibody (dilution 1:50). Note the two distinct bands at 63 kDa (arrow). **(B)** Blocked antibody with twice (2x) the amount of the synthetic peptide (1:50). **(C)** Blocked antibody with 10-fold (10x) the amount of the synthetic peptide. **(D)** Unblocked anti-arthropsin antibody (1:500). Note the two distinct bands at 63 kDa (arrow). **(E)** Blocked antibody with twice (2x) the amount of the synthetic peptide (1:500). **(F)** Blocked antibody with 10-fold (10x) the amount of the synthetic peptide (1:500). Note the absence of specific bands in all experiments with blocked anti-arthropsin antibody **(B,C,E,F)**.

To test the specificity of the anti-arthropsin serum, we have performed western blot experiments on both, lysates of the onychophoran brain (**Figure 6**) as well as on the recombinant expressed protein (**Figure 7**). In the western blot of protein extract from brains of several specimens of *E. rowelli*, all lanes represent different dilutions of the lysate (**Figure 6**). Distinct bands occur around 55 kDa in all lanes. The lanes 1 and 3 show an additional weak band at 38 kDa and lane 1 displays a weak third band at 28 kDa (**Figure 6**). In western blot experiments with the recombinant expressed protein, distinct bands occur at 63 kDa in both loaded samples – the bacterial cell lysate and the purified protein at different dilutions (**Figures 7A,D**; see Materials and Methods section for additional details). The control experiment with the blocked anti-arthropsin antibody revealed no signal (**Figures 7B,C,E,F**).

## Discussion

Our Western blot data revealed an affinity of the anti-arthropsin antibody to arthropsin of *E. rowelli* (**Figures 6** and **7**). Both the determined molecular mass of arthropsin around 55 kDa in the tissue lysate (**Figure 6**) as well as the determined molecular mass of the MBP-fused protein of 63 kDa (truncated Er-arthropsin [20 kDa] + MBP [42.5 kDa]; **Figures 7A,D**) correspond to their expected molecular masses. The molecular mass of Er-arthropsin is consistent with the molecular masses of opsins (Terakita, 2005). Although the additional bands in lanes 1 and 3 (**Figure 6**) might be due to a degradation of the protein during the experiment, these results, in conjunction with the absence of signal in samples with the blocked anti-arthropsin antibody (**Figures 7B,C,E,F**), suggest that the newly generated anti-arthropsin antibody is able to specifically bind to arthropsin of the onychophoran *E. rowelli*.

**FIGURE 8:**
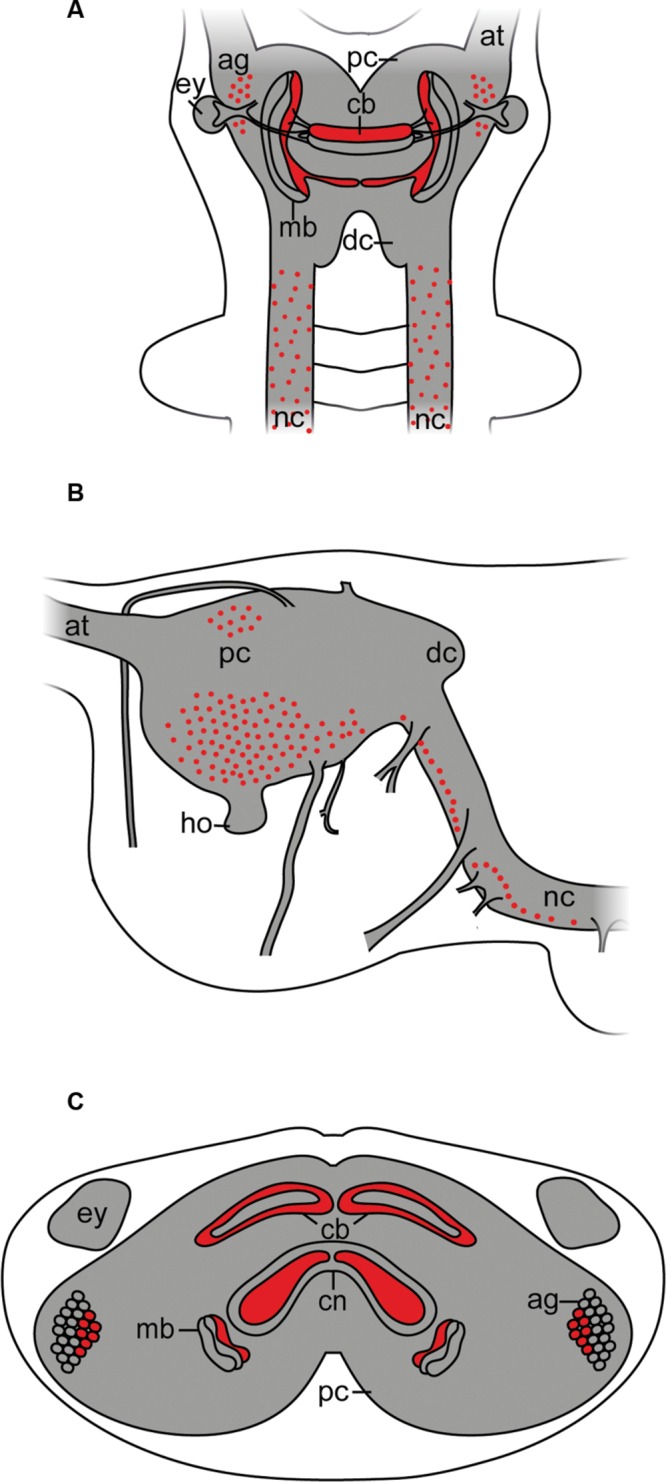
**Simplified diagram illustrating the position of Er-arthropsin immunoreactive somata and neuropils in the anterior nervous system of Onychophora.** Immunoreactivity within the nerve cord neuropils and the antennal tracts not shown. **(A)** Dorsal view. Immunoreactive somata and neuropils within the brain are shown. **(B)** Lateral view. Immunoreactive somata within the brain are shown. Immunoreactive neuropils are not shown. **(C)** Transverse view (based on cross sections of the head). Immunoreactive neuropils within the brain and immunoreactive somata within the antennal glomeruli are shown. Abbreviations: ag, antennal glomeruli; at, antennal tract; cb, central body; cn, central neuropil; dc, deutocerebrum; ey, eye; ho, hyprocerebral organ; mb, mushroom body; nc, nerve cord; pc, protocerebrum.

Although arthropsins and r-opsins have a common evolutionary origin (cf. **Figure 1**; Hering and Mayer, 2014), our data show that arthropsin of the onychophoran *E. rowelli* is expressed in a divergent pattern from onychopsin, the only known onychophoran r-opsin (Hering et al., 2012; Eriksson et al., 2013; Beckmann et al., 2015). While onychopsin occurs exclusively in the photoreceptor layer of the eye (Beckmann et al., 2015), arthropsin is expressed in the central nervous system, including the brain and the ventral nerve cords (**Figure 8**). This is in line with previous reverse transcription PCR data, which revealed that arthropsin is expressed in the central nervous system but not in the eyes of the spider *Cupiennius salei* and the onychophoran *E. kanangrensis* (see Colbourne et al., 2011; Eriksson et al., 2013; Futahashi et al., 2015).

Interestingly, the expression pattern of arthropsin in *E. rowelli* resembles that of the (most likely non-visual) c-opsin rather than onychopsin, as both are expressed in a ventral group of protocerebral neurons as well as in the connecting cords (Beckmann et al., 2015). The similarities in the expression pattern between the onychophoran c-opsin and arthropsin suggest that arthropsin might be a non-visual rather than visual opsin. Nevertheless, arthropsin might still be a photosensitive protein that is involved, for example, in the circadian clock, as has been shown for other opsins (Panda et al., 2002; Katti et al., 2010).

Our data further revealed that arthropsin is expressed in the mushroom bodies and the antennal/olfactory lobes within the brain of *E. rowelli*. The mushroom bodies are the most prominent neuropils in the brains of various arthropods, onychophorans, and some vagile polychaetous annelids (Loesel and Heuer, 2010; Mayer, 2016). Between these taxa are striking similarities in the morphology of the mushroom bodies, as they typically consist of lobed neuropils with long, parallel axons (Strausfeld et al., 1998, 2006; Farris and Sinakevitch, 2003; Loesel and Heuer, 2010; Mayer, 2016). The mushroom bodies of onychophorans show a characteristic structure with calyces, which are innervated from dendrites, a pair of pedunculi, which connect the mushroom bodies to the central body, and three longitudinal lobed neuropils (Schürmann, 1995; Strausfeld et al., 2006; Mayer, 2016).

The function of the onychophoran mushroom bodies is unknown. In insects, homonymous structures are responsible for temporal integration of sensory signals; they mediate learning and memory processes and receive olfactory input from the antennal lobes (de Belle and Heisenberg, 1994; Schürmann, 1995; Heisenberg, 1998). In fact, it has been shown that the mushroom bodies of *Drosophila melanogaster* play a key role in associative learning of olfactory information (Laurent and Naraghi, 1994; Bose et al., 2015). In the honeybee *Apis mellifera* and fruitfly *D. melanogaster* olfactory sensory neurons with olfactory receptors are present in the antenna and converge into a few glomeruli in the antennal lobe and synapse with specific second-order neurons, especially the mushroom bodies (Tanaka et al., 2004; Kirschner et al., 2006; Zube et al., 2008; Galizia and Rössler, 2010; Ramdya and Benton, 2010).

Neuroanatomical evidence suggests that the mushroom bodies of onychophorans are also second-order neuropils of the olfactory pathway (Strausfeld et al., 1998). The olfactory neurons of the antenna in *A. mellifera* transmit olfactory information to higher brain centers, including the mushroom bodies and the lateral protocerebrum (Kirschner et al., 2006). Therefore, based on the localization of arthropsin in the antennal lobes of *E. rowelli*, we speculate that this protein might play a role in olfactory signal processing. Interestingly, arthropsin is expressed only in the median lobe of each mushroom body in *E. rowelli*, suggesting that there must be a functional difference between the individual lobes of the mushroom body in Onychophora. This finding and the fact that the olfactory glomeruli are an outgrowth of the mushroom bodies (de Belle and Heisenberg, 1994; Strausfeld et al., 1998) support our hypothesis that arthropsin might be involved in the olfactory processes in *E. rowelli* and that it may function as a non-visual opsin.

## Conclusion

Our data suggest that a potential functional change might have occurred in the lineage of opsins from a non-visual role to a visual function. The expression of Er-arthropsin in the mushroom bodies and the antennal lobes of *E. rowelli* indicate a putative role of this protein in olfactory processing as a non-visual opsin, as has been shown for G-protein coupled receptors in some arthropods (Galizia and Rössler, 2010). In insects, the antennal lobes act as the first-order neuropils, which receive signal from the olfactory receptors; the signal is then transferred from the antennal lobes to the mushroom bodies, which thus function as the second-order neuropils (Tanaka et al., 2004). A similar organization has been described from onychophorans (Strausfeld et al., 1998, 2006), suggesting that a similar transduction pathway might exist in these animals. The basal position of arthropsin in the opsin tree and its potential role in olfactory processing in Onychophora are in line with the idea that olfaction might be the oldest sensory interaction with the external environment (Jones and Reed, 1989). Given that opsin proteins are old molecules, their ancestral role might generally not have been in animal vision.

## Author Contributions

IS, LH, and GM designed the experiments, carried out research, and wrote the manuscript.

## Conflict of Interest Statement

The authors declare that the research was conducted in the absence of any commercial or financial relationships that could be construed as a potential conflict of interest.

## Footnotes

1 http://www.biotech.ou.edu/
